# Importance of oxidative stress in the evaluation of acute pulmonary embolism severity

**DOI:** 10.1186/s12890-022-02076-x

**Published:** 2022-10-17

**Authors:** Gülseren Sagcan, Dildar Konukoglu, Hafize Uzun, Orhan Arseven, Gulfer Okumus, Caglar Cuhadaroglu

**Affiliations:** 1 Department of Respiratory Medicine, Acibadem Medical Faculty, Acibadem University, Istanbul, Turkey; 2 Department of Medical Biochemistry, Cerrahpasa Medical Faculty, Istanbul University-Cerrahpasa, Istanbul, Turkey; 3 Department of Medical Biochemistry, Faculty of Medicine, İstanbul Atlas University, Istanbul, Turkey; 4 Department of Respiratory Medicine, İstanbul Medical Faculty, İstanbul University, Istanbul, Turkey

**Keywords:** Pulmonary thromboembolism, Ischemia-modified albumin, Advanced protein oxidation products, Total antioxidant capacity, Pro-oxidant-antioxidant balance

## Abstract

**Background:**

Pulmonary embolism (PE) is a common and potentially life-threatening disorder. Our study was aimed to investigate whether oxidative stress markers can be used as clinical markers in the evaluation of acute PE (APE) severity.

**Methods:**

47 patients with objectively documented diagnosis of APE were recorded. Of these patients, 14 had low-risk PE, 16 had moderate-risk PE, and 17 had high-risk PE. 21 healthy subjects were also enrolled in this study. Ischemia-modified albumin (IMA), prooxidants-antioxidants balance (PAB), advanced protein oxidation products (AOPPs), and ferric reducing antioxidant power (FRAP) were measured as oxidative stress parameters to evaluate the role of oxidative stress.

**Results:**

In the low-risk and moderate-risk APE groups, AOPPs and PAB levels were significantly higher and FRAP levels were significantly lower than those in the control group. AOPPs and IMA levels in the patients with high-risk PE were significantly higher than those in both the low-risk and moderate-risk APE patients. There was a significant correlation between levels of AOPPs and the levels of both IMA (r: 0.462, *p* < 0.001) and PAB (r:0.378, *p* < 0.005). Serum FRAP levels were negatively correlated with PAB (r:− 0.683, *p* < 0.001) and AOPPs levels (r:− 0,384, *p* < 0.001). There was also a significant positive correlation between the serum IMA and PAB levels.

**Conclusions:**

We clearly demonstrated that reactive oxygen species formation is significantly enhanced in APE. IMA and AOPPs may be used as clinical markers in the evaluation of APE severity in clinical practice. However, further studies with larger patient populations and longer follow-up periods are required to confirm the mechanisms underlying these findings.

## Introduction

Pulmonary embolism (PE) is a relatively common cardiovascular emergency. By occluding the pulmonary arterial bed, it may lead to acute life-threatening situations. PE is a difficult diagnosis that may be missed because of its non-specific clinical presentation [1].

On the other hand, acute PE (APE) is a life-threatening disease leading to reperfusion of previously ischemia of the lung parenchyma. Pulmonary infarction occurs with the development of hemorrhagic necrosis in the lung parenchyma distal to the pulmonary artery occluded in PE [2]. Oxidative stress accompanies this phenomenon. Previous studies have proven that hypoxia-reoxygenation and ischemia-reperfusion cause oxidative stress accompanied by the production of oxygen free radicals exceeding the endogenous antioxidant capacity [3, 4]. In addition, in the case of ischemia, an albumin molecule called ischemia-modified albumin (IMA) is formed as a result of structural changes in the last amino terminal that binds metal in the serum albumin structure [5]. The increase in IMA concentration is currently used in the evaluation of patients with coronary syndrome as a marker of myocardial ischemia [6]. There are also studies evaluating IMA measurements in the diagnosis of PE [7, 8].

In our clinical research, it was aimed to investigate the changes in oxidative and antioxidant markers such as IMA, advanced oxidation protein products (AOPPs), prooxidants-antioxidants balance (PAB) and ferric reducing antioxidant power (FRAP) levels in patients diagnosed with APE were classified as high-risk, moderate-risk, low-risk before the initiation of anticoagulant / thrombolytic therapy.

## Materials and methods

 The study protocol was carried out by the ethical guidelines of the 1975 Declaration of Helsinki and the permission of the Institutional Scientific and Human Research Ethics Committee of the Istanbul Medical Faculty. Forty seven patients with PE (22 male, 25 female; mean age: 56.7 ± 15.42 years) and 21 healthy subjects (11 male, 10 female; mean age: 57.14 ± 10.59 years) were enrolled in the study. Each subject provided written informed consent and was informed clearly about the details of the study and blood sampling.

Twenty-one healthy volunteers with no previous history of disease and normal physical examination findings were recruited into the control group and included in the study. APE was diagnosed by spiral CT angiography in all 47 patients with suspected PE by clinical and laboratory evaluation. Data were collected on the clinical and laboratory findings of the patients at diagnosis including arterial blood gas values and chest roentgenograms, the presence of underlying diseases or predisposing factors for pulmonary thromboembolism, the results of all diagnostic procedures including ECG, echocardiography, nuclear imaging studies, right heart catheterization, and pulmonary angiography. Both the simplified Well’s and the modified Geneva scores were used for clinical scoring [9, 10].

Cardiopulmonary resuscitation, hemodynamic instability (systolic blood pressure < 90 mmHg or a drop in systolic blood pressure by > 40 mmHg for > 15 min with signs of end-organ hypoperfusion), inotrope use, recurrent or diffuse APE, development of lower limb thrombosis, the use of mechanical ventilation within one month of admission, newly developed chest pain and dyspnea, and bleeding episode due to anticoagulant use were regarded as the adverse events [11]. All consecutive patients with suspicious clinical symptoms (acute onset dyspnea, chest pain, tachycardia, and tachypnea with the need for oxygen supplementation) associated with APE admitted to the emergency department were prospectively evaluated. All patients were stratified into risk classes according to the simplified Pulmonary Embolism Severity Index (sPESI) (high- and low-risk) and the algorithm proposed by the European Society of Cardiology (ESC) 2019 guidelines [12]. Severity assessment was carried out classifying patients into four groups: low risk (LR), intermediate risk (IMR), and high risk (HR). The principal criteria for categorizing PE as high risk (HR) were arterial hypotension and cardiogenic shock. Arterial hypotension was defined as a systolic arterial blood pressure < 90 mm Hg or a drop in systolic arterial blood pressure of at least 40 mm Hg for at least 15 min. Shock was defined as manifestation of tissue hypoperfusion and hypoxia, including an altered level of consciousness, oliguria or cool, clammy extremities. Intermediate risk (IMR) patients had preserved systemic arterial pressure and right ventricular dysfunction. Low risk (LR) patients had normal both systemic blood pressure and right ventricular function. Of our 47 patients, 14 (29.7%) had LR, 16 (34%) had IMR and 17 (%36.2) had HR.

Exclusion criteria of the patient group were as follows: (1) Other acute ischemic diseases such as acute coronary syndrome, acute ischemic cerebrovascular disease, acute peripheral arterial occlusion, or acute mesenteric ischemia newly diagnosed when questioned during the admission to the emergency department, (2) Advanced liver, kidney or heart failure, (3) PE treatment already initiated, (4) Refusal to participate in the study. Patients who died or were hospitalized before they applied to the emergency department and completed the necessary procedures, and patients who did not have sufficient information in the hospital information system were not included in the study. The exclusion criteria applied during the enrollment of the healthy control group were similar to those in the patient group. Also, healthy subjects did not have any endocrine, vascular, cardiac or inflammatory diseases were chosen as the control group.

### Sample collection and preparation

Blood samples were taken from the brachial vein by using the venipuncture technique at the time of presentation. Vacutainer tubes without anticoagulants were used to obtain serum. Serum specimens were obtained after 15 min of centrifugation at 3000 rpm. Specimens to be used for measuring oxidative stress markers were pipetted into Eppendorf tubes and stored at − 80 °C.

### Measurements of the concentrations of plasma advanced protein oxidation products (AOPPs)

Spectrophotometric determinations of AOPPs concentrations were performed using a modification of the Gelisgen et al. method [13]. The coefficients of intra- and inter-assay variation were 3.1% (*n* = 20) and 3.5% (*n* = 20), respectively.

### ***Measurement of serum ferric reducing antioxidant power (FRAP) concentrations***

The FRAP assay was performed according to the protocol of Benzie and Strain [14], with minor modifications. The coefficients of intra- and inter-assay variations were 4.2% (*n* = 20) and 5.1% (*n* = 20), respectively.

### Measurement of serum prooxidant-antioxidant balance (PAB) concentrations

The PAB assay was performed according to the method of Alamdari et al. [15] with minor modifications. The coefficients of intra- and inter-assay variation were 5.0% (*n* = 20) and 6.1% (*n* = 20), respectively.

### Measurement of serum ischemia-modified albumin (IMA) concentrations

IMA concentration was assessed by a modification of the Bar-Or et al. method [6]. The coefficients of intra- and inter-assay variations were 4.1% (*n* = 20) and 5.2% (*n* = 20), respectively.

The other biochemical parameters were measured using routine methods with commercial kits and an autoanalyzer.

### Statistical analysis

Statistical analysis was performed using SPSS 17.0 version for Windows Statistical Program (SPSS, Chicago, IL, USA). All data were expressed as means ± standard deviation (SD). Power analysis was performed to determine the sample size, α = 0.05 β = 0.20, 1 − β = 0.80 and the power of the test was calculated as 0.80. Descriptive statistics were obtained, and data were tested for normality using the Kolmogorov-Smirnov test for Gaussian distribution. To compare the parameters of the various patients group, first the nonparametric Kruskal–Wallis test. The particular groups were compared by the Mann-Whitney U test. For correlation analysis, Spearman’s Rho Correlation Coefficient was determined. Values of *p* < 0.05 were considered significant.

## Results

General and clinical characteristics of PE patients grouped into three categories were given in Table 1. The high-risk PE group had significantly lower platelet count, serum albumin and protein levels when compared to both the low-risk (*p* < 0.05, *p* < 0.05 and *p* < 0.01) and moderate-risk PE groups (for each *p* < 0.05).

**Table 1:** General and clinical characteristics of patients with acute pulmonary embolism (APE)

	Controls (*n*:21)	Low-risk (*n*:14)	Intermediate-risk (*n*:16)	High-risk (*n*:17)	*p*
Gender (M/F)	11/10	7/7	4/12	11/6	–
BMI (mm^2^/kg)	25.34 ± 2.51	27.83 ± 5.54	29.99 ± 4.11	27.87 ± 4.95	> 0.05
Hypertension (*n*)	–	5	8	7	
Diabetes mellitus (*n*)	–	2	3	1	
COPD (*n*)	–	3	1	5	
Immobilization (*n*)	–	10	10	12	
Trauma (*n*)	–	0	2	0	
Malignancy (*n*)	–	4	5	6	
Pregnancy (*n*)	–	1	1	1	
Major general surgery (*n*)	–	3	5	5	
Pulse rate per minute	–	94.79 ± 10.8	87.88 ± 14.19	98.71 ± 19.41	< 0.05
Number of breaths per minute	–	22.21 ± 5.12	23.75 ± 4.91	25.18 ± 5.26	< 0.05
Systolic blood pressure (mmHg)	120 ± 8.15	119.29 ± 11.91	120.31 ± 14.66	92.65 ± 13.71	< 0.05
Diastolic blood pressure (mmHg)	82.34 ± 3.64	72.86 ± 8.25	75.31 ± 9.57	59.71 ± 11.79	< 0.01
C-reactive protein (mg/dL)	1.87 ± 0.09	44.64 ± 39.16	54.62 ± 43.93	55.04 ± 44.73	< 0.01
Leukocyte counts ( /mm³)	7896 ± 1498	8945 ± 3812	9281 ± 3466	9730 ± 4815	< 0.05
Hemoglobin (g/dL)	12.46 ± 1.74	12.44 ± 1.5	10.89 ± 3.3	11.81 ± 2.76	> 0.05
Hematocrit (%)	41.85 ± 3.76	35.73 ± 4.22	34.24 ± 5.53	35.98 ± 8.27	> 0.05
Platelet counts ( /mm³)	325.87 ± 48.98	273.79 ± 93.67	288.5 ± 164.3	199.29 ± 91.27	< 0.01
Albumin (g/dL)	4.12 ± 0.34	3.59 ± 0.29	3.52 ± 0.45	3.01 ± 0.91	> 0.05
Total protein (g/dL)	7.83 ± 0.2	6.76 ± 0.35	6.67 ± 0.4	6.36 ± 0.33	> 0.05
NT-proBNP (pg/mL)	35.72 ± 24.65	75.21 ± 67.2	274.32 ± 490.56	809.76 ± 705.12	< 0.001
cTnT (ng/mL)	0 ± 0.01	0 ± 0.01	0.03 ± 0.09	0.04 ± 0.09	> 0.05

*
COPD*, chronic obstructive pulmonary disease; *NT-proBNP*, N-terminal prohormone of brain natriuretic peptide

The serum oxidative stress marker levels of the groups were given in Table 2. In the low-risk and moderate-risk PE groups, serum AOPPs and PAB levels were significantly higher (*p* < 0.001 and *p* < 0.001; *p* < 0.001 and *p* < 0.001) and FRAP levels were significantly lower (*p* < 0.001; *p* < 0.001) than those in the control group. There was no significant difference between the low-risk and moderate-risk PE groups concerning the oxidative marker levels. Patients with high-risk PE had significantly higher AOPPs, IMA and PAB (for each *p* < 0.001) and significantly lower FRAP level (*p* < 0.001) than controls. AOPPs and IMA levels were significantly higher in the patients with high-risk PE when compared to both the low-risk (*p* < 0.001 and *p* < 0.001) and moderate-risk PE patients (*p* < 0.01 and *p* < 0.05). Although the mean serum PAB level was higher in the high-risk PE group when compared to both the moderate-risk and high-risk PE groups, differences did not reach statistically significant levels.

**Table 2:** Serum advanced protein oxidation products (AOPPs), ferric reducing antioxidant power (FRAP), pro-oxidant-antioxidant balance (PAB) and ischemia modified albumin (IMA) levels of the patients with pulmonary embolism (PE) and the controls

	Controls (*n*:21)	Low-risk (*n*:14)	Intermediate-risk (*n*:16)	High-risk (*n*:17)	*p*
IMA (U/mL)	10.43 ± 1.96	10.12 ± 2.23	10.87 ± 2.67	14.66 ± 5.54	< 0.01
AOPPs (µM chloramine T)	39.6 ± 6.8	50.7 ± 13.4	57.7 ± 18.8	81.4 ± 22.9	< 0.001
FRAP (M uric acids)	0.20 ± 0.03	0.10 ± 0.03	0.10 ± 0.03	0.11 ± 0.05	> 0.05
PAB (% H_2_O_2_)	40.2 ± 3.9	89.1 ± 26.7	96.2 ± 19.9	105.3 ± 21.8	< 0.01

There was a significant correlation between serum AOPPs level and both IMA (r: 0.462, *p* < 0.001) and PAB levels (r: 0.378, *p* < 0.005). Serum FRAP level was negatively correlated with PAB (r: − 0.683, *p* < 0.001) and AOPPs levels (r: − 0,384, *p* < 0.001). There was also a significant positive correlation between serum IMA and PAB levels (r: 0.380, *p* < 0.005).

## Discussion

Increasing evidence from both experimental and clinical studies has suggested that oxidative stress plays a major role in the pathogenesis of PE [16, 17]. Pulmonary vascular resistance (PVR) increases as a result of mechanical obstruction in the vessels and vasoconstriction caused by inflammatory mediators and hypoxia. The blood that passes through the lungs without gas exchange with the alveoli is called an intrapulmonary shunt. The progression of pulmonary hypertension and the intrapulmonary shunt circulation induce an arterial hypoxia and the reduction of oxygen saturation. It is widely known that hypoxia-reoxygenation and ischemia-reperfusion induce oxidative stress with a concomitant imbalance between the production of oxygen free radicals and endogenously available antioxidants [18, 19]. We studied the levels of oxidative stress markers named IMA, PAB, AOPPs and FRAP in PE and their roles in the determination of the severity of PE. Under acute ischemic conditions, the metal binding capacity of albumin to transition metals such as copper, nickel and cobalt is reduced, generating a metabolic variant of the protein referred to as IMA [20] IMA levels in the patients with high-risk PE were significantly higher when compared to those in both the low-risk and moderate-risk PE patients. “IMA” is one of the earliest markers of ischemia. These data represent our preliminary results, and we continue to follow our patients with measuring these parameters with six-month intervals to see the status of ischemia. There are several human and experimental studies about the diagnostic value of IMA in the diagnosis of PE, which were conducted by Türedi et al. and published in the literature [7–22]. They showed that the level of IMA might be of use in the diagnosis of PE. The authors also suggested that IMA was a good alternative to D-dimer in PE diagnosis regarding both the cost and the efficiency. However, D-dimer may be a more relevant marker than IMA which has been proposed as a new marker in the biochemical determination of severity of PE based on radiological findings [7]. Hogg et al. [23] evaluated the ability of the IMA assay to diagnose DVT and PE in a prospective cohort. They found that excluding these patient groups marginally increased the AUC for IMA and IMA/albumin in the diagnosis of PE (excluding the patients with extreme values) The investigators believe that if a new diagnostic marker is found, it should not only be simple to use but also be used on all patients, not just a small patient subset. On the other hand, another study of theirs [24] showed that IMA levels are not specific for deaths related with PE. In comparison, no such relation was detected in this study, probably due to the severity of the right ventricular dysfunction.

Our clinical investigation revealed that PE had led to increased IMA levels that had been augmented following PE severity. IMA may also be an end-product of tissue ischemia. Further studies are required to confirm the mechanisms underlying this effect.

AOPPs, which are proteins damaged by the oxidative stress, most notably albumin and its aggregates, have recently begun to attract the attention of various investigators [25]. In the general population, plasma level of AOPPs was found as an independent risk factor for coronary artery disease (CAD) [26], whereas high plasma AOPPs was found to be related with atherosclerotic cardiovascular events in nondiabetic patients with chronic kidney disease (CKD) [27] Ours is the first study to investigate whether there is a correlation between PE severity and AOPPs. In all patient groups, AOPPs levels were significantly higher when compared to the control group. AOPPs level in the patient group with high-risk PE was also significantly higher than that of both the low-risk and moderate-risk PE groups. There was a significant correlation between serum level of AOPPs and both IMA and PAB levels. In the present study, a novel marker (AOPPs assay) was analyzed for providing information about the level of oxidative damage in the proteins found in plasma for determining the severity of PE. The obtained results corroborate the IMA and PAB data.

Oxidative stress is an imbalance between the production of prooxidants and antioxidant defenses, being in favor of prooxidants. It is usually related to the increased formation of reactive oxygen species (ROS) and is considered to play a pivotal role in the pathogenesis and development of ischemia and its complications [28] The PAB assay may be able to provide valuable information regarding the oxidant-antioxidant status. Likewise, to the best of our knowledge, the serum PAB method employed in the present study has not been previously used to investigate the oxidant-antioxidant status in PE. In the low-risk and moderate-risk PE groups, PAB levels were significantly higher than that of the control group. Although the mean serum PAB level was higher in the high-risk PE group than those in the moderate-risk and low-risk PE groups, the differences were not able to reach statistically significant levels. There was also a significant positive correlation between serum IMA and PAB levels. Therefore, our study and the other studie [29–31] confirm that the PAB levels are increased in patients with CAD. We suggest that the PAB assay may be useful for a CAD risk predictor in diseases such as PE. It may also help to identify patients with high levels of oxidative stress, may be useful in the early diagnosis of vascular diseases and early interventions. Further studies are required to elucidate the contribution of PAB to PE severity.

The effect of the prooxidant or the antioxidant molecules in serum is additive. Various methods for the separate measurement of the total oxidant or antioxidant status have been proposed. For the evaluation of the prooxidant-antioxidant balance, we assayed the PAB and FRAP levels with the purpose of determining both the oxidant and the antioxidant status. FRAP can be defined as the cumulative action of all antioxidants present in the serum, thus providing a composed parameter rather than the sum of measurable antioxidants [14]. In our study, all patients had significantly lower FRAP levels than controls. There was no significant difference in oxidative marker levels between patients with low-risk and moderate-risk PE. Serum FRAP levels were negatively correlated with PAB and AOPPs levels. Sousa-Santos et al. [32] investigated whether pretreatment with tempol (a general ROS scavenger) prevented matrix metalloproteinase (MMP) activation and protected against cardiomyocyte injury of acute pulmonary thromboembolism (APT). They also investigated the possible therapeutic effects of tempol administration after APT. They showed that antioxidant treatment might prevent MMP activation and might protect against cardiomyocyte injury after APT. Neto-Neves et al. [33] demonstrated that pretreatment with atorvastatin protected against pulmonary hypertension associated with APT and that sildenafil improved this response. These findings may reflect antioxidant effects and inhibited neutrophils/MMPs activation. Clinical studies should be carried out to validate the beneficial effects exerted by this combination of drugs during APT. The validation of the total antioxidant capacity for monitoring the efficiency of antioxidant supplementation during PE should be further investigated.

In this study, NT-proBNP was found to be the most potent predictor of unfavorable outcomes in APE. This finding may be regarded as evidence for the presence of right ventricular dysfunction in the study group.

### Limitation of study

Although this study is prospective and seeks to answer an important clinical question, the study has its own limitations. Chief among these is the lack of information about whether treatments that may have normalized vital values were given, and if so, what these treatments were. Again, the fact that the sample size is not large enough and that it is a single-centered study can be counted among the limitations of the study.

We clearly demonstrated that ROS formation is significantly enhanced in PE. Since they are also measured by a rapid and low-cost technique, we suggest that IMA and AOPPs may be used as clinical markers in the evaluation of PE severity in clinical practice. Although serum IMA levels are affected by many physiological variables such as exercise and diseases such as heart failure, renal failure or liver failure, determination of serum IMA levels may be helpful to clinicians in cases in which the selection of treatment or imaging method is required. However, further studies with larger patient populations and longer follow-up periods are required to confirm the mechanisms underlying these findings.

## Data Availability

The datasets used and/or analysed during the current study available from the corresponding author on reasonable request.
